# A systematic review of the applications of markerless motion capture (MMC) technology for clinical measurement in rehabilitation

**DOI:** 10.1186/s12984-023-01186-9

**Published:** 2023-05-02

**Authors:** Winnie W. T. Lam, Yuk Ming Tang, Kenneth N. K. Fong

**Affiliations:** 1 Department of Rehabilitation Sciences, The Hong Kong Polytechnic University, Kowloon, Hong Kong SAR China; 2 Department of Industrial and Systems Engineering, The Hong Kong Polytechnic University, Kowloon, Hong Kong SAR China

**Keywords:** Markerless motion capture, Clinical measurement, Rehabilitation

## Abstract

**Background:**

Markerless motion capture (MMC) technology has been developed to avoid the need for body marker placement during motion tracking and analysis of human movement. Although researchers have long proposed the use of MMC technology in clinical measurement—identification and measurement of movement kinematics in a clinical population, its actual application is still in its preliminary stages. The benefits of MMC technology are also inconclusive with regard to its use in assessing patients’ conditions. In this review we put a minor focus on the method’s engineering components and sought primarily to determine the current application of MMC as a clinical measurement tool in rehabilitation.

**Methods:**

A systematic computerized literature search was conducted in PubMed, Medline, CINAHL, CENTRAL, EMBASE, and IEEE. The search keywords used in each database were “Markerless Motion Capture OR Motion Capture OR Motion Capture Technology OR Markerless Motion Capture Technology OR Computer Vision OR Video-based OR Pose Estimation AND Assessment OR Clinical Assessment OR Clinical Measurement OR Assess.” Only peer-reviewed articles that applied MMC technology for clinical measurement were included. The last search took place on March 6, 2023. Details regarding the application of MMC technology for different types of patients and body parts, as well as the assessment results, were summarized.

**Results:**

A total of 65 studies were included. The MMC systems used for measurement were most frequently used to identify symptoms or to detect differences in movement patterns between disease populations and their healthy counterparts. Patients with Parkinson’s disease (PD) who demonstrated obvious and well-defined physical signs were the largest patient group to which MMC assessment had been applied. Microsoft Kinect was the most frequently used MMC system, although there was a recent trend of motion analysis using video captured with a smartphone camera.

**Conclusions:**

This review explored the current uses of MMC technology for clinical measurement. MMC technology has the potential to be used as an assessment tool as well as to assist in the detection and identification of symptoms, which might further contribute to the use of an artificial intelligence method for early screening for diseases. Further studies are warranted to develop and integrate MMC system in a platform that can be user-friendly and accurately analyzed by clinicians to extend the use of MMC technology in the disease populations.

## Introduction

Markerless motion capture (MMC) technology has been developed to avoid the need for marker placement during tracking and analyzing human movement [1]. By elimination of the time-consuming marker placement procedure, motion capturing experiment can be performed in a more convenient way [2]. Without the constraints that brought by body markers on movement, the development of MMC technology allows the capture of a more lifelike human motion in the environment, in a more natural way, and with the feature that it uses more portable and low-cost sensors compared to marker-based multi-camera systems [3], MMC in turn creates the potential of additional applications.

Previous studies have been conducted to compare the accuracy of MMC and body-marker-based analysis systems. Bonnechere et al. [4] compared the measuring accuracy of full body scanning by Microsoft Kinect 3D scanner software versus that of a high-resolution stereophotogrammetric system, which is a marker-based system in the healthy population. They concluded that Kinect is a reliable markerless tool that is suitable for use as a fast estimator of morphology. Schmitz et al. [5] validated the accuracy of Kinect in measuring knee joint angle of a jig by comparing its measurement using a digital inclinometer that acted as a ground-truth, and they reported that the performance of the Kinect system was satisfactory in terms of knee flexion and abduction. The accuracy of using a smartphone as a measurement system for joint angle has been reviewed by Mourcou et al. [6], who concluded that smartphone applications are reliable for clinical measurements of joint position and range of motion (ROM).

Earlier in 2006, Mündermann et al. [7] described several methods of MMC video processing modules including background separation, visual hull, and iterative closest point methods, etc., and pointed out that MMC has the potential to achieve a level of accuracy that facilitates the biomechanics research of normal and pathological human movement. Together with the reliable performance of MMC technology in the measurement of joint angle and body movement as reflected by [5, 6], it is suggested that the MMC system can be further applied to the rehabilitation field to measure patients’ motor function. However, the actual application of MMC technology for clinical measurement in rehabilitation is still at a preliminary stage. Most of the extant studies have focused on calibration of the MMC system or on validating the MMC system only on healthy persons. Applied research on the actual use of MMC technology in measurements in patient groups has been very diverse: Vivar and the teams [8] applied MMC technology in people with Parkinson’s disease (PD) to detect and classify their tremor level, while Gritsenko et al. [9] used Kinect as the MMC system to measure the shoulder ROM for women breast cancer patients after surgery. Instead of applying MMC technology in adults, Chin et al. [10] assessed the level of proprioceptive ability in children with cerebral palsy by using Kinect as the MMC system to measure the arm position of both healthy children and children with unilateral spastic cerebral palsy (USCP). These researchers found significant differences between the proprioceptive ability of the typically developing children and the children with USCP, as measured by Kinect, thus suggesting that MMC technology has the potential to be useful as a clinical measurement tool for proprioception.

Despite these trials, however, studies on the applications of MMC technology in clinical evaluation are still preliminary and limited in number, and it remains inconclusive how MMC technology can benefit therapists, patients, or the healthcare system, in terms of measuring patients’ conditions. Review studies have been conducted on the use of MMC technology in rehabilitation training, but not in regard to its use in clinical measurement including application of MMC technology in clinical assessment and detection of kinematic parameters that assist in disease diagnosis [11]. Mousavi Hondori and Khademi [12] reviewed the clinical impact of Kinect in rehabilitation, but their study did not cover other types of MMC technology. Therefore, to investigate the current uses of MMC technology as an assessment tool in the healthcare field, in this review we put less focus on the engineering components and attempted primarily to determine the current evidence for using MMC as a measurement tool, in order to further explore the potential benefits of MMC technology in rehabilitation evaluations. In this paper, we define clinical measurement as identification and measurement of movement kinematics in a clinical population [13], while MMC technology include systems and methods that capture and analysis movements without the need of marker placement, including video-based analysis. This systematic review further investigated: (1) the types of patients to whom MMC technology has been applied; (2) the contents of the MMC measurements; (3) the types of MMC systems used; and (4) the efficacy of these MMC systems as measurement tools.

## Methods

### Search strategy

A systematic computerized literature search was conducted by one of the authors (WTL) in PubMed, Medline, CINAHL, CENTRAL, EMBASE, and IEEE. Only peer-reviewed articles were included. The search keywords used in each database were “Markerless Motion Capture OR Motion Capture OR Motion Capture Technology OR Markerless Motion Capture Technology OR Computer Vision OR Video-based OR Pose Estimation AND Assessment OR Clinical Assessment OR Clinical Measurement OR Assess.” A manual search was also conducted that included searching Google Scholar using the same keywords, and the reference lists of the previous systematic reviews were also screened. The published data were not limited, and the last search took place on March 6, 2023.

### Inclusion criteria

Studies were included if they met certain inclusion criteria. Specifically, the studies had to: (1) be peer-reviewed; (2) apply MMC technology for measurement; (3) involve subjects with symptomatic conditions; (4) have any quantitative study design except systematic reviews; (5) include at least one assessment item for clinical evaluation; and (6) be published in English.

### Exclusion criteria

Studies were excluded if they met any one of the following exclusion criteria: (1) studying only healthy persons; (2) focusing only on calibration of the MMC system; (3) applying MMC technology only in rehabilitation training; or (4) not reporting results of an assessment evaluation.

### Data extraction

The information we assessed included: (1) the types of MMC systems used in the studies; (2) the conditions of the participants that underwent the measurement, such as diagnoses or disabilities; and (3) the contents of the measurements conducted. The interpretations of the studies’ results were extracted and are presented in a summary table (Table 1). The contents of the measurement included the body functions or body parts that were measured, and the context in which the assessment was conducted.

**Table 1:** Details of the selected studies

Study	Patient types	Sample size (n)	MMC system	Measurement items	Content of measurement	Context of measurement	Primary results	Results interpretation
Cho et al. 2009 [28]	PD	Patients with PD (7); healthy controls (7)	Sony HDR-HC3 camcorder	Gait pattern	Recognition of PD gait by algorithm combining PCA with LDA	Laboratory	The proposed system can identify healthy adults and patients with PD by their gaits with high reliability	Video-based analysis helps in discriminating the gait patterns of PD patients and healthy adults
Adde et al. 2010 [41]	CP	Infants with high risk of CP (30)	Digital video camera	Quantity of motion, velocity and acceleration of the centroid of motion	Comparison of quantity of motion and centroid of motion in infants who developed into CP with those who did not develop into CP	Hospital	Quantity of motion mean, median, and standard deviation were significantly higher in the group of infants who did not develop CP than in the group who did develop CP	Quantitative variables related to the variability of the center of infant movement and to the amount of motion predicted later CP in young infants with high sensitivity and specificity
Bahat et al. [61]	Chronic neck pain	Patients with chronic neck pain (25); asymptomatic participants (42)	Customized VR assessment system	Cervical ROM (flexion, extension, rotation, and lateral flexion)	Comparison of cervical movement in patients with chronic neck pain, versus in healthy controls	Laboratory	Significant group differences for 3 of the kinematic measures: V_peak_, V_mean,_ and number of velocity peaks	“Goal-directed fast cervical movements performed by patients with chronic neck pain were characterized by lower velocity and decreased smoothness compared with asymptomatic participants” [61]
Chen et al. 2011 [29]	PD	Patients with PD (12); healthy adults (12)	CCD video camera	Gait parameters including gait cycle time, stride length, walking velocity, and cadence	Quantification of gait parameters	Structured environment	KPCA-based method achieved a classification accuracy of 80.51% in identifying different gaits	Kinematic data extracted from video might allow clinicians to obtain the quantitative gait parameters and assess the progression of PD
Khan et al. 2013 [14]	PD	Patients diagnosed with advanced PD (13); healthy controls (6)	Video recordings, analyzed by CV algorithm	Index-finger motion in finger tapping, features including speed, amplitude, rhythm, and fatigue in tapping were computed	SVM classification to categorize the patient group between UPDRS-FT symptom severity levels, and to discriminate between PD patients and healthy controls	Medical facility	The proposed CV-based SVM scheme discriminated between control and patient group with an average of 94.5% accuracy	The ML framework offers good classification performance in distinguishing symptom severity levels based on clinical ratings, as well as in identifying PD patients and the healthy controls
Lowes et al. 2013 [65]	Dystrophinopathy	Patients with dystrophinopathy (5); healthy controls (5)	Kinect	Upper extremity functional reaching volume, velocity, and rate of fatigue	Validity and Reliability of the MMC system in capturing upper extremity functional reaching volume, movement velocity, and rate of UE fatigue in individuals with dystrophinopathy	Laboratory	Preliminary test-retest reliability of the MMC method for 2 sequential trials was excellent for functional reaching volume	“The newly available gaming technology has potential to be used to create a low-cost, accessible, and functional upper extremity outcome measure for use with children and adults with dystrophinopathy” [65]
O’Keefe et al. 2013 [60]	FXS	Males with FXS (13); healthy controls (7)	BioStage™	Motion parameters (frequency and total traveled distance) of body segments during 30 s of story listening while standing in the observation space	Comparison between groups, MMC system results were compared with scores on video-capture methodology and behavioral rating scales	Laboratory	Arm and foot travel distances were significantly greater in the FXS group compared with the controls	“Motion parameters obtained from the markerless system can quantify increased movement in subjects with FXS relative to controls” [60]
Olesh et al. 2014 [46]	Stroke	Patients with stroke (9)	Kinect	10 movements of the upper extremity	Quantitative scores derived from motion capture were compared to qualitative clinical scores produced by trained human raters	Laboratory	Strong linear relationship was found between qualitative scores and quantitative scores derived from both standard and low-cost motion capture system	“The low-cost motion capture combined with an automated scoring algorithm is a feasible method to assess objectively upper-arm impairment post stroke” [46]
Gritsenko et al. 2015 [9]	Breast cancer	Women with mastectomy (4) or lumpectomy (16) for breast cancer	Kinect	Active and passive shoulder motions	Regression coefficients for active movements were used to identify participants with clinically significant shoulder ROM limitation	Laboratory	Participants had good ROM in the shoulder ipsilateral to the breast surgery at the time of testing. Three participants showed clinically significant shoulder motion limitations	Findings support the use of MMC approach as part of the automated screening tool to identify people who have shoulder motion impairment
Lee et al. 2015 [64]	AC of shoulder	Healthy volunteers (15); patients with AC (12)	Kinect	Shoulder ROM	Validity of measure shoulder ROM in AC by calculating the agreement of Kinect measurements with measurements obtained using a goniometer, and assessment of its utility for the diagnosis of AC	Laboratory	Measurements of the shoulder ROM using Kinect showed excellent agreement with those taken using a goniometer	“Kinect can be used to measure shoulder ROM and to diagnose AC as an alternative to a goniometer” [64]
Tupa et al. 2015 [30]	PD	Patients with PD (18); healthy age-matched individuals (18); students (15)	Kinect	Leg length, normalized average stride length, and gait velocity	A two-layer sigmoidal neural network was used for the classification of gait features (stride length and gait velocity)	Laboratory	Results showed high classification accuracy for the given set of individuals with PD and the age-matched controls	Kinect has potential to be used in the detection of abnormal gait and the recognition of PD
Sá et al. 2015 [56]	Schizophrenia	Clinically stable outpatients with schizophrenia (13); healthy controls (16)	BioStage™	Kinematic parameters and motor patterns during a functional task	Comparison of the kinematic parameters and motor patterns of patients with schizophrenia and those of healthy subjects	Laboratory	Patients with schizophrenia displayed a less developed movement pattern during performance of overarm throwing	“The presence of a less mature movement pattern can be an indicator of neuro-immaturity and a marker for atypical neurological development in schizophrenia” [56]
Kim et al. 2016 [47]	Stroke	Patients with hemiplegic stroke (41)	Kinect	Upper extremity motion of 13 of 33 items of upper extremity motor FMA	Correlation of the prediction accuracy for each of the 13 items between real FMA scores and scores using Kinect were analyzed	Laboratory	Prediction accuracies ranged from moderate to good in each item. Correlations were high for the summed score for the 13 items between real FMA scores and scores obtained using Kinect	“Kinect can be a valid way to assess upper extremity function, which may be useful in the setting of unsupervised home-based rehabilitation” [47]
Matsenet al. 2016 [75]	Variety of diagnoses (cuff disease, instability, arthritis)	Patients with a variety of diagnoses, including cuff disease, instability, arthritis (32); control healthy subjects (10)	Kinect	Shoulder active ROM	Correlation of Kinect shoulder active ROM measurement with SST	Laboratory	The total SST score was strongly correlated with the range of active abduction. The ability to perform each of the individual SST functions was strongly correlated with active motion	“Kinect provides a clinically practical method for objective measurement of active shoulder motion” [75]
Chin et al. 2017 [10]	CP	Children with USCP (31); typically developing children (21)	Kinect v2	Proprioception	Comparison of proprioceptive ability in children with USCP versus that in typically developing children	Laboratory	Children with USCP showed significant impairments in proprioception compared with typically developing children	The use of MMC technology can clearly identify differences in proprioceptive ability between typically developing children and children with UCSP
de Bie et al. 2017 [63]	ALS	Patients diagnosed with ALS (10)	Kinect	Upper extremity reachable workspace RSA	Evaluation of longitudinal changes in upper extremity reachable workspace RSA versus the ALSFRS-R, ALSFRS-R upper extremity sub-scale and FVC	Laboratory	RSA measures were able to detect changes in the upper limbs while the ALSFRS-R could not. The RSA measures were also able to detect a declining trend similar to that of FVC	“Kinect-measured RSA can detect declines in upper extremity ability with more granularity than current tools” [63]
Bakhti et al. 2018 [48]	Stroke	Individuals with hemiparetic stroke (19)	Kinect	Movements of 25 predefined body “joints” that approximately correspond to the center of the anatomical joint or body part	Use of ICC and linear regression analysis to quantify the degree to which an ultrasound 3D motion capture system motion capture system and Kinect measurements were related	Laboratory	PANU scores determined by the Kinect were similar to those determined by the ultrasound 3D motion capture system	“The Kinect sensor can accurately and reliably determine the PANU score in clinical routine” [48]
Bonnechère et al. 2018 [49]	Stroke	Healthy young adults (40); elderly adults (22); and patients with chronic stroke (10)	Kinect	Parameters including length, angle, velocity, angular velocity, volume, sphere, and surface of upper limb motion	The different scores and parameters were compared for the three groups	Laboratory	Highly significant differences were found for both the shoulders’ total angle, the velocity for young adults and elderly individuals, and patients with stroke	Results of the evaluation could be useful in monitoring patients’ conditions during rehabilitation, while further studies are needed to select which parameters are the most relevant
Butt et al. 2018 [15]	PD	Participants with PD (16); healthy people (12)	LMC	PSUP, OPCL, THFF, and POST	Comparison of parameters between a PD group and control group; Supervised learning methods SVM, LR, and NB for classification of patients with PD and healthy subjects	Laboratory	The best performing classifier was the NB. All the other subset features selected by the other feature selection methods, showed the worst classification performance in all ML classifiers (LR, NB, SVM)	“LMC is not yet able to track motor dysfunction characteristics from all MDS- UPDRS proposed exercises” [15]
Dranca et al. 2018 [31]	PD	Patients with PD (30)	Kinect	Gait step, limbs angle, and bent angles related to Parkinson disease	Classification of different PD stages by the features from FoG using classification algorithms	Hospital	The accuracy obtained for a particular case of a Bayesian Network classifier built from a set of 7 relevant features is 93.40%	“Using Kinect is adequate to build an inexpensive and comfortable system that classifies PD into three different stages related to FoG” [31]
Li et al. 2018 [25]	PD	Patients with PD (9)	Consumer grade video camera	416 features including kinematics, frequency distribution extracted from 14 joint angle positions	Quantifying the severity of levodopa-induced dyskinesia by video-based features	Laboratory	Features achieved similar or superior performance to the UDysRS for detecting the onset and remission of dyskinesia	“The proposed system provides insight into the potential of computer vision and deep learning for clinical application in PD ” [25]
Li et al. 2018 [32]	PD	Patients with PD after DBS (24)	Ordinary 2D video camera	TUG sub-task segmentation	Frame classification algorithm to classify video frame in sub tasks of TUG test	Semi-controlled environments	Classification accuracies for the sub-tasks ‘Walk,’ ‘Walk-Back,’ and ‘Sit-Back’ are apparently higher than that of the other three sub-tasks	The results support that clinical parameters for the assessment of PD can be automatically acquired from TUG videos
Martinez et al. 2018 [26]	PD	Patients with PD (6); healthy subjects (6)	DARI system	BME of 16 different movements	UPDRS-III and BME of 16 different movements in six controls paired by age and sex were compared with those in PD populations with DBS in ‘on’ and ‘off’ states	Laboratory	A better performance in the BME was correlated with a lower UPDRS-III score. No statistically significant difference between patients in ‘on’ and ‘off’ states of DBS regarding BME	The DARI MMC system is accurate in PD classification
Pantzar-Castilla et al. 2018 [45]	CP	Participants with CP (18)	Kinect 2 for Xbox One	Gait variables (i.e., Knee flexion at initial contact; Maximum knee flexion at loading response; Minimum knee flexion in stance; Maximum knee flexion in swing)	Comparison of 2D MMC and 3D marker-based gait analysis methods for the selected variables	Laboratory	The reliability within 2D Markerless and 3D gait analysis was mostly good to excellent	2D MMC is a convenient tool that could be used to assess the gait in children with CP
Rammer et al. 2018 [67]	Pediatric manual wheelchair users	Pediatric manual wheelchair users (30)	Kinect 2.0	Upper extremity kinematics during manual wheelchair propulsion (i.e., joint range of motion and musculotendon excursion)	Kinematic parameters were used to develop and evaluate a markerless wheelchair propulsion biomechanical assessment system	Laboratory	Inter-trial repeatability of spatiotemporal parameters, joint range of motion, and musculotendon excursion were all found to be significant	“A markerless wheelchair propulsion kinematic assessment system is a repeatable measurement tool for pediatric manual wheelchair users” [67]
Langevin et al. 2019 [16]	PD	Patients with PD (127); healthy controls (127)	Webcam	Frequencies of hand movement in hand motor task	Comparison of the differences in the hand motion between the groups with and without PD	Home Setting	PD group had a mean frequency that is lower than the control group in the hand motor tasks	“Online framework that assesses features of PD could be introduced during a clinic visit to initially supplement the tool with personal support” [16]
Lee et al. 2019 [17]	PD	Participants with PD that are receiving benefit from DBS (8)	LMC	PSUP, OPCL, and THFF tasks during ‘on’ and ‘off’ condition, amplitude, frequency, velocity, slope, and variance were extracted from each movement	Correlation of the kinematic features with the overall bradykinesia severity score (average MDS-UPDRS ratings across tasks)	Laboratory	An exhaustive LOSOCV assessment identified PSUP, OPCL, and THFF as the best task combination for predicting overall bradykinesia severity	“Data obtained from the LMC can predict the overall bradykinesia severity in agreement with clinical observations and can provide reliable measurements over time” [17]
Liu et al. 2019 [18]	PD	Patients with PD (60)	Camera	Periodic pattern of hand movements in finger tapping, hand clasping and hand pro/supination	Correlation analysis on each feature parameter and clinical assessment scores; Classification of bradykinesia	Semi-controlled environment	Classification accuracy in 360 examination videos is 89.7%	Reliable assessment results on Parkinsonian bradykinesia can be produced from video with minimal device requirement
Sato et al. 2019 [33]	PD	Patients with PD (117 in phase I and 2 in phase II); healthy controls (117)	Home video camera	Cadence , gait frequency, gait speed, step length, step width, foot clearance	Estimation of cadence of periodic gait steps from the sequential gait features using the short-time pitch detection approach	Structured environment	Cadence estimation of gait in its coronal plane in the daily clinical setting was successfully conducted in normal gait movies using ST-ACF	2D movies recorded with a home video camera is helpful in identifying an effective gait and calculate its cadence in normal and pathological gaits
Vivar et al. 2019 [8]	PD	Patients with PD (20)	LMC	Tremor levels measured during hand extension and pushing the ball action	Classification of tremor level in PD according to the MDS-UPDRS standard	Laboratory	The proposed method classified the patient measurements following MDS-UPDRS in tremor levels 0, 1, and 2 with high accuracy	“It is possible to classify the different levels of tremor in patients with PD using only two statistical features, such as homogeneity and contrast” [8]
Caruso et al. 2020 [52]	ASD	Infants with high risk of ASD (50); infants with low risk of ASD (53)	Video recording	Quantity of motion, centroid of motion, presence of repetitive movements in the motion of limbs	Kinematic parameters related to upper and lower limb movements in infants with low risk and high risk of ASD	Bed	Early developmental trajectories of specific motor parameters were different in high-risk infants later diagnosed with neurodevelopmental diseases from those of infants developing typically	“Computer-based analysis of infants’ movements may support and integrate the analysis of motor patterns of infants at risk of neurodevelopmental diseases in research settings” [52]
Chambers et al. 2020 [66]	Neuromotor disease	Infants at risk of neuromotor impairment (19); healthy infants (85)	GoPro cameras, YouTube video	Absolute position and angle, variability of posture, velocity of movement, variability of movement, complexity, left-right symmetry of movement	Extent of kinematic features from infants at risk deviate from the group of healthy infants as reflected by Naïve Gaussian Bayesian Surprise metric	Childcare facility, hospital, natural environment	Infants who are at high risk for impairments deviate considerably from the healthy group	“Markerless tracking promises to improve accessibility to diagnostics, monitor naturalistic movements, and provide a quantitative understanding of infant neuromotor disorders” [66]
Fujii et al. 2020 [70]	Patients with gait disturbance	Patients with gait ataxia (6); control subjects (6)	Kinect 2, migrated to Azure Kinect	Gait parameters (e.g., walking speed and stride length)	Gait comparison between the patient group and the healthy subject group	Laboratory	Significant differences were observed between the patient group and the healthy subject group in terms of the mean value and variation of stride length	“A low-cost noninvasive motion capture device can be used for the objective clinical assessment of patients with stroke and PD who display manifestations of gait and motor deficits” [70]
Hu et al. 2020 [34]	PD	Patients with PD (45)	Video	Gait parameters, motion patterns	Automatic FoG detection by fine-grained human action recognition method	Structured environment	The experimental results demonstrate the superior performance of the proposed method over the state-of-the-art methods	“Anatomic joint graph representation provides clinicians an intuitive interpretation of the detection results by localizing key vertices in a FoG video” [34]
Krasowicz et al. 2020 [42]	CP	Patients with diagnosed ICP (8)	4DBODY system	TMFPI developed based on movement sequences	TMFPI compared with the assessment made according to the GMFM-88 scale	Laboratory	The system provided results agreeable with the clinical indicator GMFM-88 and with clinical observations of a PT	“The conducted assessments indicated that the use of dynamic 3D surface measurements is a promising direction of research and can provide valuable information on patient movement patterns” [42]
Lin et al. 2020 [19]	PD	Patients with PD (121)	iPhone 6s Plus	Motor behaviors, including stability, completeness, and self-similarity	Quantification of motor behaviors in patients with PD and bradykinesia recognition by a periodic motion-based network consisting of an autoencoder and fully connected neural network	Laboratory	The proposed periodic motion model delivers the F-score of 0.7778 for bradykinesia recognition	Using single RGB video for bradykinesia recognition is easy and convenient for patients and doctors
Oña et al. 2020 [39]	PD	Patients with PD (20)	LMC	Manual dexterity in BBT	Evaluation the validity of VR-BBT to reliably measure the manual dexterity	Laboratory	VR-BBT significantly correlated with the conventional assessment of the BBT	“VR-BBT could be used as a reliable indicator for health improvements in patients with PD” [39]
Pang et al. 2020 [20]	PD	Patients with PD; healthy controls (22)	Logitech HD Pro C920 webcams	Hand motion in tap thumb to the finger, creating a fist, pronation and supination of hand and resting state	Measurement of parkinsonian symptomology using automated analysis of hand gestures	Structured environment	Behavior of patients with PD and control subjects can be distinguished by analyzing the detailed motion features of their hands/fingers	Automatic hand movement detection method may help clinicians to identify tremor and bradykinesia in PD
Sabo et al. 2020 [58]	Dementia	Older adults with dementia (14)	Kinect	Gait parameters including cadence, average and minimum margin of stability per step, average step width, coefficient of variation of step width and time, the symmetry index of the step times, number of steps in the walking bout	Correlation and regression of gait features with clinical scores UPDRS and SAS	Hospital	Gait features extracted from both 2D and 3D videos are correlated to UPDRS-gait and SAS-gait scores of parkinsonism severity in gait	“Vision-based systems have the potential to be used as tools for longitudinal monitoring of parkinsonism in residential settings” [58]
Schroeder et al. 2020 [43]	CP	High-risk infants (29)	Kinect v1	Infants’ general movement	Correlation of expert GMA ratings of standard RGB videos with GMA ratings on SMIL motion videos of the same sequence	Clinical environment	GMA based on computer-generated virtual 3D infant body models closely corresponded to the established gold standard based on conventional RGB videos	SMIL motion video might capture the movement characteristics required for GMA of infants
Williams et al. 2020 [21]	PD	Patients with PD (20); control participants (15)	Smartphone	Bradykinesia assessed by finger tapping	ML models to predict no/slight bradykinesia or mild/moderate/severe bradykinesia, and presence or absence of Parkinson’s diagnosis	Clinical setting	SVM with radial basis function kernels predicted presence of mild/moderate/ severe bradykinesia with good accuracy. NB model predicted the presence of PD with moderate accuracy	The proposed approach supports the detection of bradykinesia without purchasing extra hardware devices
Williams al. 2020 [22]	PD	Patients with idiopathic PD (39); healthy controls (30)	Smartphone	Bradykinesia assessed by finger tapping	Correlation of machine learning models with clinical ratings of bradykinesia	Clinical setting	Computer measures correlated well with clinical ratings of bradykinesia	“The research provides a new tool to quantify bradykinesia. It could potentially be used to support diagnosis and monitoring of PD” [22]
Zefinetti et al. 2020 [62]	SCI patients using a wheelchair	Patients with SCI (60)	Kinect v2	Kinematic data, including humeral elevation, horizontal abduction of humerus, humeral rotation, elbow flexion, trunk flexion/extension of wheelchair propulsion	Correlation between the movements and the patients’ assessment	Laboratory	The measurements computed by the proposed system showed a good reliability for analyzing the movements of SCI patients’ wheelchair propulsion	“The proposed markerless solutions are useful for an adequate evaluation of wheelchair propulsion” [62]
Abbas et al. 2021 [57]	Schizophrenia	Patients with Schizophrenia (18); healthy controls (9)	Smartphone	Head movement	Comparison of head movement measurements between patients and healthy controls, relationship of head movement to schizophrenia symptom severity	Home setting/ Natural environment	Rate of head movement in participants with schizophrenia and those without differed significantly; head movement was a significant predictor of schizophrenia diagnosis	“Remote, smartphone- based assessments were able to capture meaningful visual behavior for computer vision-based objective measurement of head movement” [57]
Ardalan et al. 2021 [71]	Neurodevelopmental Disorders	Children with 16p11.2 mutation (15); TD children (12)	A single point-and-shoot camera	Gait synchrony, balance parameters	Comparison of gait synchrony and balance in children with 16p11.2 mutation and TD children	Natural environment	Children with 16p11.2 mutation had significantly less whole-body gait synchrony and poorer balance compared to TD children	Remote video analysis approach facilitates the research in motor analysis in children with developmental disorders
Cao et al. 2021 [35]	PD	Patients with PD (18); healthy controls (42)	RGB camera	Shuffling step	Detection of shuffling step and severity assessment	Hospital	3D convolution on videos achieves an average shuffling step detection accuracy of 90.8%	Video-based detection method might facilitate more frequent assessment of FoG in a more cost-effective way
Hurley et al. 2021 [69]	Patients awaiting TKR who were attending POAC	Patients awaiting unilateral primary TKR (23)	BioStage™	LLM, VVM	Comparison of LLM and VVM performed clinically, radiologically, and using MMA	Laboratory	Discrepancies existed in LLM and VVM when evaluated using clinical, radiological, and MMA modalities	The MMC system should not be the only method to assess the patients for TKR
Kojovic et al. 2021 [55]	ASD	Children with ASD (169); TD children (68)	2D camera	Patterns of atypical postures and movements	Differentiation between children with ASD and TD using non-verbal aspects of social interaction by deep neural network	Clinical setting	The classification accuracy is 80.9% with the prediction probability positively correlated to the overall level of symptoms of autism in social affect and repetitive and restricted behaviors domain	Remote machine learning-based ASD screening might be possible in the future
Lee et al. 2021 [50]	Stroke	Patient with stroke (206)	Smartphone	Swing time asymmetry between paretic and non-paretic lower limbs while walking	Classification of dependence in ambulation by employing a deep model in 3D-CNN	Hospital	The trained 3D-CNN performed with 86.3% accuracy, 87.4% precision	“Monitoring ambulation using videos may facilitate the design of personalized rehabilitation strategies for stroke patients with ambulatory and balance deficits in the community” [50]
Li et al. 2021 [23]	PD	Patients with PD (157)	Video	Skeleton sequence from finger-tapping test	Classification of finger tapping performance according to MDS-UPDRS score	Hospital	Fine-grained classification net- work achieved an accuracy of 72.4% and an acceptable accuracy of 98.3%	Vision-based assessment method has potential for remote monitoring of PD patients in the future
Mehdizadeh et al. 2021 [59]	Dementia	Individuals admitted to a specialized dementia inpatient unit (54)	Kinect v2	Gait variables, including gait stability, step length, step time variability, step length variability	Changes in quantitative gait measured over a period during a psychogeriatric admission	Laboratory	Results showed that there was deterioration of gait in this cohort of participants, with men exhibiting greater decline in gait stability	“Quantitative gait monitoring in hospital environments may provide opportunities to intervene to prevent adverse events, decelerate mobility decline, and monitor rehabilitation outcomes” [59]
Negin et al. 2021 [53]	ASD	Children with or without ASD (108)	YouTube video	Spinning, head banging, hand action, arm flapping	Recognition of ASD associated behaviors	Natural environment	HOF descriptor achieves the best results when used with MLP classifier	“An action-recognition-based system can be potentially used to assist clinicians to provide a reliable, accurate, and timely diagnosis of ASD disorder” [53]
Nguyen-Thai et al. 2021 [44]	CP	Videos of infants who were at 14–15 weeks post-term age (235)	Smartphone	FM	Predicted the risk of CP by FM	Natural environment	Pose sequences were strong signals that retained motion information of joints and limbs while ignoring irrelevant, distracting visual artifacts	A STAM model can be used to identify infants at risk of cerebral palsy via video-based infant movement assessment
Rupprechter et al. 2021 [36]	PD	Patients with PD (729)	Smartphone	Leg ratio difference, vertical angle of the body, horizontal angle of the ankles and wrists, horizontal distance between the heels, speed of the ankles, step frequency	Estimation of severity of gait impairment in Parkinson’s disease using a computer vision-based methodology	Hospital and offices	Step frequency point estimates from the Bayesian model were highly correlated with manually labelled step frequencies	“Automated systems for quantifying Parkinsonian gait have great potential to be used in combination with, or the absence of, trained assessors, during assessments in the clinic or at home” [36]
Stricker et al. 2021 [37]	PD	Patients with PD (24)	Standard camera	Step length	Reliability of step length measurements from 2D video in patients with stroke; comparison of the step lengths of patients with/without a recent history of falls	Structured environment	Step length measurements from the video demonstrated excellent intra- and inter-rater reliability; patients with PD who had experienced a fall within the previous year demonstrated shorter step lengths	“Quantification of step length from 2D video may offer a feasible method for clinical use” [37]
Wei et al. 2021 [68]	Wheelchair user	Full-time wheelchair users (91)	Kinect	Wheelchair transfer motions including joint angles and positions	ML algorithm for evaluation of the quality of independent wheelchair sitting pivot transfers	Structured environment	Accuracies of the ML classifier were over 71%	“The results show promise for the objective assessment of the transfer technique using a low cost camera and machine learning classifiers” [68]
Williams et al. 2021 [72]	Tremor	Patients with PD (9); patients with essential tremor (5); patient with functional tremor (1)	Smartphone	Hand tremor at rest and in posture	Measurement of hand tremor frequency	Clinical setting	There was less than 0.5 Hz difference between the computer vision and accelerometer frequency measurements in 97% of the videos	“The study suggests a potential new, contactless point-and-press measure of tremor frequency within standard clinical settings, research studies, or telemedicine” [72]
Wu et al. 2021 [40]	PD	Patients with PD (7)	LMC	Hand kinematic in finger tapping hand opening and closing, and hand pronation and supination	Quantification of the motor component of bradykinesia	Laboratory	Average velocity and average amplitude of pronation/supination isolate the bradykinetic feature	“The LMC achieved promising results in evaluating PD patients’ hand and finger bradykinesia” [40]
Ferrer-Mallol et al. 2022 [73]	DMD	Patients with DMD (8)	Smartphone	Time, pattern of movement trajectory, smoothness and symmetry of movement	Quantitative measurement of the motor performance of the patients in the functional tasks	Home	Computer vision analysis allowed characterization of movement in an objective manner	“Video technology offers the possibility to perform clinical assessments and capture how patients function at home, causing minimal disruption to their lives” [73]
Guo et al. 2022 [24]	PD	Patients with PD (48); healthy controls (11)	RGB camera	Finger movement in finger tapping test	Classification of PD from finger tapping video	Structured environment	Classification accuracy is of 81.2% on a newly established 3D PD hand dataset of 59 subjects	Novel computer-vision approach could be effective in capturing and evaluating the 3D hand movement in patients with PD
Lonini et al., 2022 [51]	Stroke	Patients with stroke (8)	Digital RGB video camera	Gait parameters including cadence, double support time, swing time, stance time, and walking speed	Comparison of gait parameters obtained from clinical system and video-based method for gait analysis	Laboratory	Absolute accuracy and precision for swing, stance, and double support time were within 0.04 ± 0.11 s	“Single camera videos and pose estimation models based on deep networks could be used to quantify clinically relevant gait metrics in individuals poststroke” [51]
Morinan et al. 2022 [38]	PD	Videos from patients with PD (447)	Smartphone	Body kinematics including movement, velocity variation and smoothness	Estimation of ‘arising from chair’ task score in MDS-UPDRS	Clinical setting	Compute-vision based method can accurately quantify PD patients’ ability to perform the arising from chair action	Computer-vision based approach might be used for quality control and reduction of human error by identifying unusual clinician ratings
Vu et al. 2022 [74]	CD	Patients with CD (93)	Video recording	Peak power, frequency, and directional dominance of head movement	Quantification of oscillatory and directional aspects of HT	Structured environment	Computer-vision based method of quantification of HT exhibits convergent validity with clinical severity ratings	“Objective methods for quantifying HT can provide a reliable outcome measure for clinical trials” [74]
Morinan et al. 2023 [27]	PD	Patients with PD (628)	Consumer-grade hand- held devices	Movements during the bradykinesia examinations including finger tapping, hand movement, pronation-supination, toe tapping, leg agility	Quantification of bradykinesia according to clinician ratings	Clinical setting and laboratory	Classification model estimate of composite bradykinesia had high agreement with the clinician ratings	Computer vision technology with smartphone/ tablet devices can be adopted in the current clinical workflows
Song et al. 2023 [54]	ASD	Children with ASD (29); TD child (1)	RGB camera	Head and body movement during response to name behavior	Prediction of ASD by response to name behavior	Structured environment	The prediction method is highly consistent with clinical diagnosis	Automatic detection method can help to carry out remote autism screening in the early developmental stage of children

3D-CNN: 3D Convolutional Neural Network; AC: Adhesive Capsulitis; ALS: Amyotrophic Lateral Sclerosis; ALSFRS-R: Revised Amyotrophic Lateral Sclerosis Functional Rating Scale; ASD: Autism Spectrum Disorder; BME: Body Motion Evaluation; CCD: Commercial Digital Charge-coupled Device; CD: cervical dystonia; CP: Cerebral Palsy; CV: Computer Vision; DBS: Deep Brain Stimulation; DMD: Duchenne muscular dystrophy; FM: Fidgety Movement; FMA: Fugl-Meyer Assessment; FoG: Freezing of Gait; FoG: Freezing of gait; SAS: Simpson- Angus Scale; FVC: Forced Vital Capacity; FXS: Fragile X Syndrome; GMA: General Movement Assessment; GMFM-88: Gross Motor Function Measure-88; HOF: Histogram of Optical Flow; HT: Head Tremor; ICC: Intra-Class Correlation Coefficient; ICP: Infantile Cerebral Palsy; KPCA: Kernel-based Principal Component Analysis; LDA: Linear Discriminant Analysis; LLM: Leg Length Measurement; LMC: Leap Motion Controller; LOSOCV: Leave-One-Subject-Out Cross-Validation; LR: Logistic Regression; MDS-UPDRS: Movement Disorder Society-Sponsored Revision of the Unified Parkinson’s Disease Rating Scale; ML: Machine Learning; MLP: Multi-layer Perceptron; MMA: Markerless Motion Analysis; MMC: Markerless Motion Capture; NB: Naïve Bayes; NN: Neural Network; OPCL: Hand Opening/Closing; PANU: Proximal Arm Non-Use; PCA: Principal Component Analysis; PD: Parkinson’s Disease; PFP: Patellofemoral pain; POAC: Pre-Operative Assessment Clinic; POST: Postural Tremor; PSUP: Forearm Pronation-Supination; PT: Physiotherapist; RGB: Red Green Blue; ROM: Range of Motion; RSA: Relative Surface Area; SCI: Spinal Cord Injured; SDK: Software Development Kit; SMIL: Skinned Multi-Infant Linear Body Model; SST: Simple Shoulder Test; ST-ACF: short-time autocorrelation function; STAM: Spatio-Temporal Attention-Based Model; SVM: Support Vector Machine; TD: Typically Developing; THFF: Thumb Forefinger Tapping; TKR: Total Knee Arthroplasty; TMFPI: Trunk Mobility in the Frontal Plane Index; UDysPS: Unified Dyskinesia Rating Scale; UPDRS: Unified Parkinson’s Disease Rating Scale; UPDRS-FT: Unified Parkinson’s Disease Rating Scale-Finger Tapping; USCP: Unilateral Spastic Cerebral Palsy; VR: Virtual Reality; VVM: Varus/Valgus Knee Measurements

## Results

### Literature search and study characteristics

**Fig. 1:**
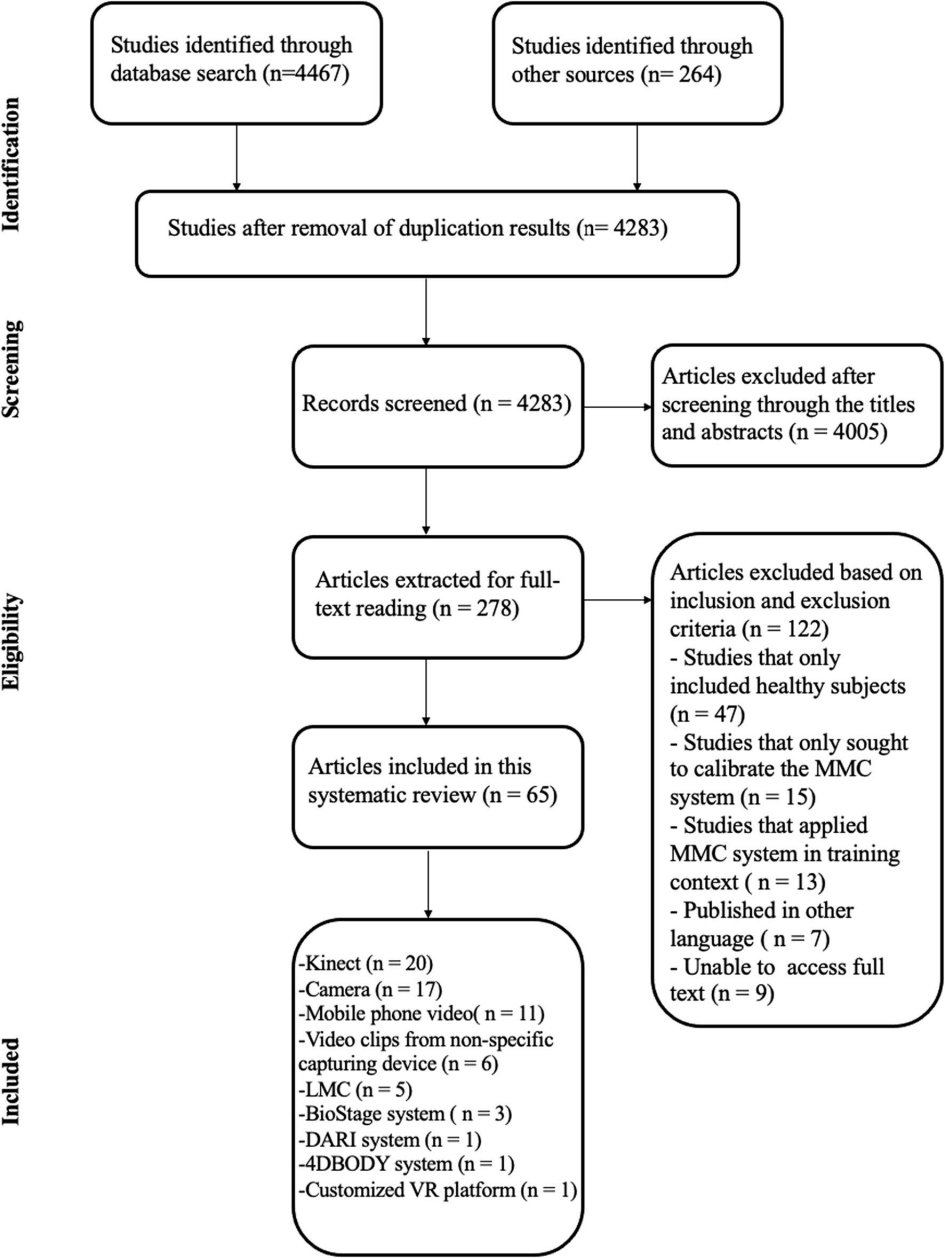
Flow chart for selection of the studies for this review

A total of 4283 articles were identified, 278 of which were selected for full-text reading after removal of duplicates and irrelevancies, according to their abstracts (Fig. 1). After next excluding 213 articles on the basis of the inclusion and exclusion criteria, 65 studies remained and were included in the final review (Fig. 1). More than 40% of the studies applied MMC technology to assess a patient population with PD (*n* = 28) [8, 14–40]. Two other diseases that had commonly been measured by the MMC system were cerebral palsy (CP) (*n* = 6) [10, 41–45] and stroke (*n* = 6) [46–51]. Four other studies focused on children with autism spectrum disorder (ASD) (*n* = 4) [52–55] while there are two studies focused on patients with schizophrenia (*n* = 2) [56, 57] and patients with dementia (*n* = 2) [58, 59] respectively. The rest of the studies were conducted on various other diseases: Fragile X syndrome (FXS) [60], chronic neck pain [61], breast cancer [9], spinal cord injury (SCI) [62], amyotrophic lateral sclerosis (ALS) [63], adhesive capsulitis of shoulder (AC) [64], dystrophinopathy [65] and neuromotor diseases [66]. There were also studies that had been conducted on wheelchair users (n = 2) [67, 68], people awaiting total knee arthroplasty (TKR) [69], patients with gait disturbance [70], patients with neurodevelopment disorders (NDD) [71], patients with tremor [72], patients with Duchenne muscular dystrophy (DMD) [73], patients with cervical dystonia (CD) [74] and patients with a variety of diagnoses [75]. Table 1 summarizes the 65 selected studies.

### Body function/body part being measured

Of the 28 studies that assessed the PD population by using MMC technology, fourteen measured the hand’s motor conditions to classify or to predict the severity of PD [8, 14–24, 39, 40]. These fourteen studies used the PD features of bradykinesia and tremor, as reflected during hand movements such as a finger-tapping exercise, to train machine-learning models to serve as classifiers. Of the remaining fourteen studies, four focused on using whole-body motion to classify PD [25–27, 38], and the other ten measured gait features to detect gait disorder in people with PD [28–37]. The measured body function for the CP population by the MMC system included gait pattern, trunk mobility, general body movement, fidgety movements, and the level of proprioceptive ability [10, 41–45]. The six studies on stroke survivors applied MMC technology to measure their upper limb movement, including their motor function, movement velocity, and joint angle [46–49] as well as lower limb movement gait parameters and walking pattern [50, 51]. The studies that worked on the ASD population mainly focused on prediction of diagnosis of ASD by children’s behavioral patterns [52–55]. The measured areas in the studies that applied MMC technology in patients with other types of diseases varied, and the details are listed in the summary table (Table 1).

### Details of measurement and efficacy

The applications of the MMC systems in measurement were classified into several categories. Sixteen out of the 65 selected studies used MMC technology in symptoms identification in disease populations [8, 14, 15, 17, 21, 25, 30–32, 36, 39, 40, 50, 53, 54, 72]. Butt et al. attempted to distinguish patients with PD from healthy subjects by features of their hand movements, reporting that their Leap Motion Controller (LMC) system together with the machine-learning models did not provide a reliable measurement for the PD symptoms [15]. Fifteen studies focused on comparing the movement patterns of the disease populations and a healthy population, with all of them reporting a significant difference in at least one of the measured parameters including gait parameters, hand movement patterns, head movement patterns and general body movements [16, 20, 26, 28, 41, 49, 52, 55–57, 60, 61, 66, 70, 71]. Fifteen studies applied MMC technology to detect and identify movement limitations or specific movement patterns of patients with certain diseases, and significant parameters that indicate movement abnormity including bradykinesia, shuffling gait, abnormal walking pattern, and tremor were identified [9, 19, 24, 29, 33–35, 37, 42–44, 51, 59, 63, 73]. Two studies used the MMC system to measure range of motion (ROM), and they suggested MMC could be an alternative to the goniometer as a tool for ROM assessment [64, 75]. Three studies used the MMC system as a tool to analyze the wheelchair propulsion skills of wheelchair users [62, 67, 68]. Ten studies correlated or compared the MMC measurements with clinical assessment scales [18, 23, 27, 38, 46–48, 58, 65, 74]. Among the other three studies, one applied MMC technology in a comparison with the optic marker system [45], one used it to measure leg length [69], and one used it as a tool to assess proprioception [10]. Only one study reported unsatisfactory results, claiming that the use of the MMC system alone to measure leg length was not accurate [69]. The details are listed in the summary table (Table 1).

### Types of MMC systems

Twenty studies used Kinect in their research, thus making Kinect the most popular MMC system used in the selected studies [9, 10, 30, 31, 43, 45–49, 58, 59, 62–65, 67, 68, 70, 75]. Sixteen studies used camera including RGB camera, digital video camera, GoPro camera and webcam [16–18, 20, 24, 25, 29, 32, 33, 35, 37, 41, 51, 54, 55, 71], while twelve studies analyzed patients’ movement by using smartphone or mobile tablet videos [14, 19, 21, 22, 27, 36, 38, 44, 50, 57, 72, 73]. Six studies performed the motion analysis from YouTube video or video recordings that captured by nonspecific capturing device [23, 34, 52, 53, 66, 74]. Five studies used the leap motion controller (LMC), an optical hand-tracking module [8, 15, 17, 39, 40]. The rest of the studies applied the BioStage™ System (Organic Motion Inc., N.Y., USA) (n = 3) [56, 60, 69]; the DARI Motion platform’s motion capture system (n = 1) [26]; the 4DBODY System (n = 1) [42], and a nonspecific customized motion capture system (n = 1) [61]. Table 2 describes and compares the characteristics of these seven types of MMC systems in terms of their mechanisms, set-up procedures, relative costs, the body part(s) that can be captured, and the systems’ methods of data extraction and analysis.

**Table 2:** Comparison of the MMC systems

MMC system	Mechanisms	Relative cost	Assessable body parts	Portability	Set-up procedure	Methods of data extraction and analysis
Kinect	Monochrome CMOS sensor and infrared projector measures player’s body by transmitting invisible near-infrared light, data are then processed by algorithms	Low	Whole body except fine hand movement	Yes	Simple	Data can be extracted by the Microsoft Kinect algorithm, and offline analysis can be performed using software such as R or MATLAB
Camera	2D images are captured directly by camera	Low	Whole body	Yes	Simple	Data is commonly analyzed by pose estimation algorithm, and kinematic features are extracted from the joint trajectories
LMC	Hand movements captured by two monochromatic IR cameras and three infrared LEDs and a rather “complex math algorithm” are used to process the raw data	Low	Hand and finger movement	Yes	Simple	Data can be obtained from the LMC SDK
BioStage™	3D images captured by high-speed color cameras and data are analyzed by computer vision software	High	Whole body	No	Complicated	The 3D motion data can be analyzed using the Motion Monitor software
Smartphone	Mobile phone camera is used to capture the movement directly	No extra cost needed	Whole body	Yes	Simple	Specific algorithms are required to analyze the video image
DARI Motion system	Uses eight high-speed cameras placed around the subject and a state-of-the-art computer-vision engine to collect whole-body data, including the fastest motions	High	Whole body	No	Complicated	Data analyzed by images captured by eight high-speed cameras using the software provided by the DARI Motion company
4DBODY System	Uses a single-frame structured light illumination method to allow the registration of the shape of body surface with a frequency of up to 120 Hz	High	Whole body	No	Complicated	Data from 4D measurement sequences can be extracted by the FRAMES software package
Customized motion capture system	Two main components: an electromagnetic tracker and an HMD. The tracker sampled motion via two sensors at 60 Hz each.	Not mentioned	Particularly neck and trunk movement	Not mentioned	Not mentioned	Tracking data can be analyzed by MATLAB software

CMOS: Complementary Metal Oxide Semiconductor, HMD: Helmet-mounted Displays, LED: Light-emitting Diode, LMC: Leap Motion Controller, SDK: Software Development Kit

## Discussion

Our results revealed that most of the research applications of an MMC system for measurement were with patient groups with physical disabilities, and more than half of the studies assessed the PD and CP populations. A possible reason for this trend could be that both PD and CP have obvious and well-defined physical signs and symptoms and abnormal movements. PD is characterized by the presence of tremor, bradykinesia, and rigidity during movement [76], whereas patients with cerebral palsy demonstrate spasticity, ataxia, rigidity in movement, and the like [77]. The characteristic types of movement in these two groups of patients might be especially favorable for detection and analysis by the MMC system because of the significant homogeneity in the patients’ movement patterns. Applications of an MMC system for measurement in other kinds of physical disabilities have been limited, and that was the case in this review, but the heterogeneous disease types that were evaluated in the selected studies suggest the possibility of a high variety of generalized uses of MMC technology in assessing different types of patients.

In addition to the use of MMC systems in applications involving physical disabilities that demonstrate observable physical symptoms, it was noteworthy that such systems were also applied in patients with mental illness and NDD, in an attempt to deduce the presence of movement markers for mental disorder and the behavior associated with NDD. Experimental use of MMC technology in patients with mental illness and NDD suggests an entirely new trend for the application of MMC technology in the clinical field. Heretofore, motion tracking has been used in targeted patients with physical disabilities, because the analysis of their movements can provide necessary information and data about their level of impairment, and that in turn can indicate their recovery progress. However, although clinical observations have demonstrated that there is a difference between the movement patterns of patients with mental illness and those of healthy individuals, application of motion capture systems to assess the physical ability of patients with mental illness is still quite limited [78]. Since traditional marker-based systems for motion analysis are time-consuming to set up given that it requires calibration procedure and attachment of markers on the body, using the traditional motion analysis marker systems might not be cost-effective to study the motion dysfunctions in patients with mental illness whose cognitive functions are predominantly affected. In fact, previous studies on motion detection of patients with mental illness adopted the fuzzy movement method, and precise actions and movement patterns have been less emphasized [79]. Therefore, the development of MMC technology allows motion capture in a more cost-effective way, and that improvement may facilitate future scientific investigations of movement patterns and motor functions in patients with mental illness. Identifying the risk of NDD by extracting the children’s behavioral features with the help of computer-vision technology also proposed a new direction of early screening of NDD [80], in which children’s developmental conditions can be closely monitored in their familiar environment without the need of attachment of markers on the infants’ body. Similarly, the studies that have applied the MMC system to compare the motion patterns of a disease population and a healthy population provide evidence for the technology’s use to identify biomarkers for certain diseases. MMC technology may also contribute to the development and use of big data for future AI screening for diseases, based on body movements. The combination of MMC technology and a machine-learning algorithm in classification of CP in infants by Nguyen-Thai et al. [44] is one of the good examples that demonstrates how MMC technology can help in the preliminary screening of diseases. Compared with screening methods for traditional diseases, which depend heavily on behavioral observations by parents or on subjective self-reported questionnaires [81], MMC technology, which identifies symptoms via movement detection, could be a more objective method for early screening for diseases, facilitating early identification of a disease and thus improving the prognosis for rehabilitation, as well as providing a tool for evaluation before and after rehabilitation.

In contrast to using MMC technology for symptoms identification or for detection of differences in movement patterns between disease groups and their healthy counterparts, other studies applied MMC technology as a direct clinical measurement tool. Although the use of the MMC system to measure leg length was found to be inaccurate [69], the use of Kinect to measure ROM was found to be reliable [64, 75]. These findings suggest that MMC technology might have the potential to serve as an alternative clinical assessment tool. MMC technology also provides a new approach to assessing functional or cognitive abilities, such as objectively evaluating proprioception, which previously relied heavily on manual evaluations by rehabilitation therapists. However, future studies on the measurement accuracy and the validity of MMC technology as a clinical measurement tool are warranted.

Microsoft Kinect, the most commonly used MMC system in the studies in this review, is a relatively low-cost, commercially available system that captures and analyzes whole-body movement. Kinect enables the capture of real-time whole body gross movements, but it appears to be less sensitive in tracking fine hand movements [82]. Although many of the studies used Kinect in their MMC measurements, the system has been out of production since 2017 and was no longer supported by the Xbox Series X, as announced by Microsoft [83]. Future rehabilitation assessors that wish to use MMC technology may have to consider using other kinds of MMC systems, or the newly developed Azure Kinect. Our review also found that the most recent studies adopted the use of camera, smartphone, or video clips from the internet in conjunction with pose estimation algorithms and motion analysis algorithm, which has been rapidly developed in the recent years, to capture images and analyze motion. Human pose estimation method is a way of identifying and classifying human joints position using computer vision, for example, the open-source libraries OpenPose and PoseNet for human pose estimation are widely adopted in motion analysis [84]. With the development of human pose estimation database containing various types of movement datasets, accuracy of pose estimation from video clips can be further enhanced by using a large set of training data. This facilitates the use computer vision methods for motion analysis in video clips captured by portable and low-cost camera rather than using specific sensors in the traditional way. The use of machine-learning algorithms allows meaningful information such as kinematic data to be extracted directly from regular videos, thus making the use of MMC technology much easier in motion capturing in a natural environment without the need to buy any extra hardware devices. Human pose estimation technology such as Convolutional Pose Machines (CPM) and convolution neural network (CNN) based methods which allow extraction of human movement information directly from video clips have been repeatedly tested by researchers [85, 86] while human pose estimation application on analyzing movement in the disease populations were reported to be useful by the studies in our review [14, 16–25, 27, 29, 32–38, 41, 44, 50–55, 57, 66, 71–74]. Given that such trajectory extraction method is in rapid evaluation and is becoming more mature for promising identification of posture [87–89], using hand-held camera or smartphone as the MMC system would be especially beneficial for understanding the motor performance of individuals in their daily living tasks, hence providing valuable information on levels of impairment and on the constraints that patients might encounter in their activities of daily living in their real-life environment. It is understandable that individuals, particularly young children and older people, might behave differently when they are placed for motion capturing in an unfamiliar laboratory or a simulated environment, thus risking the possibility that the motion analysis might not truly reflect the individuals’ actual movement patterns [90]. The use of a smartphone camera combined with an algorithm for analysis could provide a solution to that problem and suggests the feasibility of assessing patients’ daily movements through an MMC combination of a smartphone and an advanced algorithm. Since it does not require additional hardware for motion capturing, such a system would further broaden MMC technology for measurement and clinical assessment in the field of rehabilitation.

### Limitations of the current MMC technology’s applications for clinical measurement

Although the use of MMC system in motion capturing is becoming more common in movement measurement and helps us extend the application of MMC technology to clinical use, the technologies used for analyzing movement and distinguishing motor patterns are not yet generalized. Extracting and processing the data from MMC devices video files is still complicated and time-consuming, preventing the approach from being user-friendly for therapists to adopt as a quick clinical measurement tool. Further investigation is needed in order to design and develop a platform or software that can accurately analyze the movement patterns from videos in a more user-friendly and accurately way so as to further extend its application by clinicians. Although most of the studies that we included reported detecting a significant difference between the motor parameters of healthy control groups and those of disease populations, and while the identification of physical symptoms by the MMC system was also reported to be mostly effective, the sample sizes adopted by these studies were too small. A reliable AI tool for disease screening and classification will need to be trained and tested from a large set of data, to provide better specificity and sensitivity. In order to make use of MMC technology-assisted AI screening and early detection of diseases, a larger database that records movement patterns of both the disease population and the healthy population must be developed. Research on the development and selection of a suitable machine-learning or deep-learning model for classification is also needed. Ultimately, a cost-effective and accurate method for early patient screening will help therapists to identify individuals at risk and involve them in further, in-depth assessments, so that subsequent interventions can be made as early as possible. Moreover, it has been suggested that telerehabilitation could incorporate the use of MMC technology as a measurement tool for assessing and monitoring patients’ prognosis and recovery, thus offering an objective and precise evaluation of patients’ rehabilitation progress.

## Conclusions

This review explored the current uses of MMC technology to perform assessments in clinical situations. Most of the studies in the review combined MMC technology and a classification algorithm to perform symptoms identification for disease populations or to detect the differences in movement between disease groups and their healthy counterparts. Findings from these studies revealed a potential use of MMC technology for detecting and identifying disease signs and symptoms. The method might also contribute to early screening by using AI and big data to screen for diseases that lead to physical or mental disabilities. Further studies are warranted to develop and integrate MMC system in a platform that can be user-friendly and accurately analyzed by clinicians to extend the use of MMC technology in clinical measurement.

## Data Availability

Not applicable.

## References

[1] Corazza S, Mündermann L, Gambaretto E, Ferrigno G, Andriacchi TP (2010). Markerless motion capture through visual hull, articulated icp and subject specific model generation. Int J Comput Vision.

[2] Rahul M (2018). Review on motion capture technology. Glob J Comput Sci Technol.

[3] Scott B, Seyres M, Philp F, Chadwick EK, Blana D (2022). Healthcare applications of single camera markerless motion capture: a scoping review. PeerJ.

[4] Bonnechere B, Jansen B, Salvia P, Bouzahouene H, Sholukha V, Cornelis J (2014). Determination of the precision and accuracy of morphological measurements using the Kinect™ sensor: comparison with standard stereophotogrammetry. Ergonomics.

[5] Schmitz A, Ye M, Shapiro R, Yang R, Noehren B (2013). Accuracy and repeatability of joint angles measured using a single camera markerless motion capture system. J Biomech.

[6] Mourcou Q, Fleury A, Diot B, Franco C, Vuillerme N (2015). Mobile phone-based joint angle measurement for functional assessment and rehabilitation of proprioception. Biomed Res Int.

[7] Mündermann L, Corazza S, Chaudhari AM, Andriacchi TP, Sundaresan A, Chellappa R, editors. Measuring human movement for biomechanical applications using markerless motion capture. Three-dimensional image capture and applications VII; 2006: International Society for Optics and Photonics.

[8] Vivar G, Almanza-Ojeda D-L, Cheng I, Gomez JC, Andrade-Lucio JA, Ibarra-Manzano M-A (2019). Contrast and homogeneity feature analysis for classifying tremor levels in Parkinson’s disease patients. Sensors (Basel).

[9] Gritsenko V, Dailey E, Kyle N, Taylor M, Whittacre S, Swisher AK (2015). Feasibility of using low-cost motion capture for automated screening of shoulder motion limitation after breast cancer surgery. PLoS ONE.

[10] Chin K, Soles L, Putrino D, Dehbandi B, Nwankwo V, Gordon A (2017). Use of markerless motion capture to evaluate proprioception impairments in children with unilateral spastic cerebral palsy: a feasibility trial. Dev Med Child Neurol.

[11] Knippenberg E, Verbrugghe J, Lamers I, Palmaers S, Timmermans A, Spooren A (2017). Markerless motion capture systems as training device in neurological rehabilitation: a systematic review of their use, application, target population and efficacy. J Neuroeng Rehab.

[12] Mousavi Hondori H, Khademi M (2014). A review on technical and clinical impact of microsoft kinect on physical therapy and rehabilitation. J Med Eng..

[13] Sakkos D, Mccay KD, Marcroft C, Embleton ND, Chattopadhyay S, Ho ESL (2021). Identification of abnormal movements in infants: a deep neural network for body part-based prediction of cerebral palsy. IEEE Access.

[14] Khan T, Nyholm D, Westin J, Dougherty M (2013). A computer vision framework for finger-tapping evaluation in Parkinson’s disease. Artif Intell Med.

[15] Butt AH, Rovini E, Dolciotti C, De Petris G, Bongioanni P, Carboncini MC (2018). Objective and automatic classification of Parkinson disease with leap motion controller. Biomed Eng Online.

[16] Langevin R, Ali MR, Sen T, Snyder C, Myers T, Dorsey ER (2019). The PARK framework for automated analysis of Parkinson’s disease characteristics. Proc ACM Interact Mob Wearable Ubiquitous Technol.

[17] Lee WL, Sinclair NC, Jones M, Tan JL, Proud EL, Peppard R (2019). Objective evaluation of bradykinesia in Parkinson’s disease using an inexpensive marker-less motion tracking system. Physiol Meas.

[18] Liu Y, Chen J, Hu C, Ma Y, Ge D, Miao S (2019). Vision-based method for automatic quantification of Parkinsonian Bradykinesia. IEEE Trans Neural Syst Rehab Eng.

[19] Lin B, Luo W, Luo Z, Wang B, Deng S, Yin J (2020). Bradykinesia recognition in Parkinson’s disease via single RGB video. ACM Trans Knowl Discov Data.

[20] Pang Y, Christenson J, Jiang F, Lei T, Rhoades R, Kern D (2020). Automatic detection and quantification of hand movements toward development of an objective assessment of tremor and bradykinesia in Parkinson’s disease. J Neurosci Methods.

[21] Williams S, Relton SD, Fang H, Alty J, Qahwaji R, Graham CD (2020). Supervised classification of bradykinesia in Parkinson’s disease from smartphone videos. Artif Intell Med.

[22] Williams S, Zhao Z, Hafeez A, Wong DC, Relton SD, Fang H (2020). The discerning eye of computer vision: can it measure Parkinson’s finger tap bradykinesia?. J Neurol Sci.

[23] Li H, Shao X, Zhang C, Qian X (2021). Automated assessment of parkinsonian finger-tapping tests through a vision-based fine-grained classification model. Neurocomputing (Amsterdam).

[24] Guo Z, Zeng W, Yu T, Xu Y, Xiao Y, Cao X (2022). Vision-based finger tapping test in patients with Parkinson’s disease via spatial-temporal 3D hand pose estimation. IEEE J Biomed Health Inform.

[25] Li MH, Mestre TA, Fox SH, Taati B (2018). Vision-based assessment of parkinsonism and levodopa-induced dyskinesia with pose estimation. J Neuroeng Rehab.

[26] Martinez HR, Garcia-Sarreon A, Camara-Lemarroy C, Salazar F, Guerrero-González ML (2018). Accuracy of markerless 3D motion capture evaluation to differentiate between On/Off status in Parkinson’s disease after deep brain stimulation. Parkinsons Dis.

[27] Morinan G, Dushin Y, Sarapata G, Rupprechter S, Peng Y, Girges C (2023). Computer vision quantification of whole-body parkinsonian bradykinesia using a large multi-site population. NPJ Parkinson’s Dis.

[28] Cho C-W, Chao W-H, Lin S-H, Chen Y-Y (2009). A vision-based analysis system for gait recognition in patients with Parkinson’s disease. Expert Systems with applications.

[29] Chen S-W, Lin S-H, Liao L-D, Lai H-Y, Pei Y-C, Kuo T-S (2011). Quantification and recognition of parkinsonian gait from monocular video imaging using kernel-based principal component analysis. Biomed Eng Online.

[30] Tupa O, Prochazka A, Vysata O, Schaetz M, Mares J, Valis M (2015). Motion tracking and gait feature estimation for recognising Parkinson’s disease using MS Kinect. Biomed Eng Online.

[31] Dranca L, de Abetxuko Ruiz de Mendarozketa L, Goñi A, Illarramendi A, Navalpotro Gomez I, Delgado Alvarado M (2018). Using Kinect to classify Parkinson’s disease stages related to severity of gait impairment. BMC Bioinform.

[32] Li T, Chen J, Hu C, Ma Y, Wu Z, Wan W (2018). Automatic timed Up-and-Go sub-task segmentation for Parkinson’s disease patients using video-based activity classification. IEEE Trans Neural Sys Rehab Eng.

[33] Sato K, Nagashima Y, Mano T, Iwata A, Toda T (2019). Quantifying normal and parkinsonian gait features from home movies: practical application of a deep learning–based 2D pose estimator. PLoS ONE.

[34] Hu K, Wang Z, Mei S, Martens KAE, Yao T, Lewis SJG (2020). Vision-based freezing of gait detection with anatomic directed graph representation. IEEE J Biomed Health Inform.

[35] Cao X, Xue Y, Chen J, Chen X, Ma Y, Hu C (2021). Video based shuffling step detection for parkinsonian patients using 3d convolution. IEEE Trans Neural Syst Rehab Eng.

[36] Rupprechter S, Morinan G, Peng Y, Foltynie T, Sibley K, Weil RS (2021). A clinically interpretable computer-vision based method for quantifying gait in parkinson’s disease. Sensors.

[37] Stricker M, Hinde D, Rolland A, Salzman N, Watson A, Almonroeder TG (2021). Quantifying step length using two-dimensional video in individuals with Parkinson’s disease. Physiother Theory Pract.

[38] Morinan G, Peng Y, Rupprechter S, Weil RS, Leyland L-A, Foltynie T (2022). Computer-vision based method for quantifying rising from chair in Parkinson’s disease patients. Intelligence-Based Medicine.

[39] Oña ED, Jardón A, Cuesta-Gómez A, Sánchez-Herrera-Baeza P, Cano-de-la-Cuerda R, Balaguer C (2020). Validity of a fully-immersive VR-based version of the box and blocks test for upper limb function assessment in Parkinson’s disease. Sensors.

[40] Wu J, Yu N, Yu Y, Li H, Wu F, Yang Y (2021). Intraoperative quantitative measurements for Bradykinesia evaluation during deep brain stimulation surgery using Leap Motion Controller: a pilot study. Parkinson’s Disease.

[41] Adde L, Helbostad JL, Jensenius AR, Taraldsen G, Grunewaldt KH, StØen R (2010). Early prediction of cerebral palsy by computer-based video analysis of general movements: a feasibility study. Dev Med Child Neurol.

[42] Krasowicz K, Michoński J, Liberadzki P, Sitnik R (2020). Monitoring improvement in infantile cerebral palsy patients using the 4DBODY system—a preliminary study. Sensors (Basel).

[43] Schroeder AS, Hesse N, Weinberger R, Tacke U, Gerstl L, Hilgendorff A (2020). General Movement Assessment from videos of computed 3D infant body models is equally effective compared to conventional RGB video rating. Early Hum Dev.

[44] Nguyen-Thai B, Le V, Morgan C, Badawi N, Tran T, Venkatesh S (2021). A spatio-temporal attention-based model for Infant Movement Assessment from videos. IEEE J Biomed Health Inform.

[45] Pantzar-Castilla E, Cereatti A, Figari G, Valeri N, Paolini G, Della Croce U (2018). Knee joint sagittal plane movement in cerebral palsy: a comparative study of 2-dimensional markerless video and 3-dimensional gait analysis. Acta Orthop.

[46] Olesh EV, Yakovenko S, Gritsenko V (2014). Automated assessment of upper extremity movement impairment due to stroke. PloS one.

[47] Kim W-S, Cho S, Baek D, Bang H, Paik N-J (2016). Upper Extremity Functional evaluation by Fugl-Meyer Assessment Scoring using depth-sensing camera in hemiplegic stroke patients. PLoS One.

[48] Bakhti KKA, Laffont I, Muthalib M, Froger J, Mottet D (2018). Kinect-based assessment of proximal arm non-use after a stroke. J Neuroeng Rehabil.

[49] Bonnechère B, Sholukha V, Omelina L, Van Sint Jan S, Jansen B (2018). 3D analysis of upper limbs motion during rehabilitation exercises using the KinectTM sensor: development, laboratory validation and clinical application. Sensors.

[50] Lee JT, Park E, Jung T-D (2021). Machine learning-based classification of dependence in ambulation in stroke patients using smartphone video data. J Personalized Med.

[51] Lonini L, Moon Y, Embry K, Cotton RJ, McKenzie K, Jenz S (2022). Video-based pose estimation for gait analysis in stroke survivors during clinical assessments: a proof-of-concept study. Digital Biomarkers.

[52] Caruso A, Gila L, Fulceri F, Salvitti T, Micai M, Baccinelli W (2020). Early Motor Development predicts clinical outcomes of siblings at high-risk for Autism: insight from an innovative motion-tracking technology. Brain Sci..

[53] Negin F, Ozyer B, Agahian S, Kacdioglu S, Ozyer GT (2021). Vision-assisted recognition of stereotype behaviors for early diagnosis of Autism Spectrum Disorders. Neurocomputing.

[54] Song C, Wang S, Chen M, Li H, Jia F, Zhao Y (2023). A multimodal discrimination method for the response to name behavior of autistic children based on human pose tracking and head pose estimation. Displays.

[55] Kojovic N, Natraj S, Mohanty SP, Maillart T, Schaer M (2021). Using 2D video-based pose estimation for automated prediction of autism spectrum disorders in young children. Scientific Reports.

[56] Sá F, Marques A, Rocha NBF, Trigueiro MJ, Campos C, Schröder J (2015). Kinematic parameters of throwing performance in patients with schizophrenia using a markerless motion capture system. Somatosens Mot Res.

[57] Abbas A, Yadav V, Smith E, Ramjas E, Rutter SB, Benavidez C (2021). Computer vision-based assessment of motor functioning in schizophrenia: use of smartphones for remote measurement of schizophrenia symptomatology. Digital Biomarkers.

[58] Sabo A, Mehdizadeh S, Ng K-D, Iaboni A, Taati B (2020). Assessment of Parkinsonian gait in older adults with dementia via human pose tracking in video data. Journal of neuroengineering and rehabilitation.

[59] Mehdizadeh S, Faieghi M, Sabo A, Nabavi H, Mansfield A, Flint AJ (2021). Gait changes over time in hospitalized older adults with advanced dementia: predictors of mobility change. PLoS ONE.

[60] O’Keefe JA, Orías AAE, Khan H, Hall DA, Berry-Kravis E, Wimmer MA (2013). Implementation of a markerless motion analysis method to quantify hyperkinesis in males with fragile X syndrome. Gait Posture.

[61] Bahat HSPPT, Weiss PLPOT, Laufer YDPT (2010). The Effect of Neck Pain on Cervical Kinematics, as assessed in a virtual environment. Arch Phys Med Rehabil.

[62] Zefinetti FC, Vitali A, Regazzoni D, Rizzi C, Molinero G (2020). Tracking and characterization of spinal cord-injured patients by means of rgb-d sensors. Sensors (Basel).

[63] de Bie E, Oskarsson B, Joyce NC, Nicorici A, Kurillo G, Han JJ (2017). Longitudinal evaluation of upper extremity reachable workspace in ALS by Kinect sensor. Amyotroph Lateral Scler Frontotemporal Degener.

[64] Lee SH, Yoon C, Chung SG, Kim HC, Kwak Y, Park H-W (2015). Measurement of shoulder range of motion in patients with Adhesive Capsulitis using a Kinect. PLoS One.

[65] Lowes LP, Alfano LN, Yetter BA, Worthen-Chaudhari L, Hinchman W, Savage J (2013). Proof of concept of the ability of the kinect to quantify upper extremity function in dystrophinopathy. PLoS Curr..

[66] Chambers C, Seethapathi N, Saluja R, Loeb H, Pierce SR, Bogen DK (2020). Computer vision to automatically assess infant Neuromotor Risk. IEEE Trans Neural Syst Rehabil Eng.

[67] Rammer J, Slavens B, Krzak J, Winters J, Riedel S, Harris G (2018). Assessment of a markerless motion analysis system for manual wheelchair application. J Neuroeng Rehabil.

[68] Wei L, Chung C-S, Koontz AM (2021). Automating the Clinical Assessment of Independent Wheelchair sitting pivot transfer techniques. Topics Spinal Cord injury Rehab.

[69] Hurley RJ, Davey MS, Newell M, Devitt A (2021). Assessing the accuracy of measuring leg length discrepancy and genu varum/valgum using a markerless motion analysis system. J Orthop.

[70] Fujii M, Wada N, Ikeda Y, Hasegawa M, Nakazato S, Yuminaka Y (2020). Rehabilitation Assistance Systems for three-dimensional gait analysis using motion capture Devices. Advanced engineering forum.

[71] Ardalan A, Yamane N, Rao AK, Montes J, Goldman S (2021). Analysis of gait synchrony and balance in neurodevelopmental disorders using computer vision techniques. Health Informatics Journal.

[72] Williams S, Fang H, Relton SD, Wong DC, Alam T, Alty JE (2021). Accuracy of smartphone video for contactless measurement of hand tremor frequency. Movement Disorders Clinical Practice.

[73] Ferrer-Mallol E, Matthews C, Stoodley M, Gaeta A, George E, Reuben E (2022). Patient-led development of digital endpoints and the use of computer vision analysis in assessment of motor function in rare diseases. Front Pharmacol.

[74] Vu JP, Cisneros E, Lee HY, Le L, Chen Q, Guo XA (2022). Head tremor in cervical dystonia: quantifying severity with computer vision. Journal of the Neurological Sciences.

[75] Matsen FAMD, Lauder AMD, Rector KMS, Keeling PMD, Cherones AL (2016). Measurement of active shoulder motion using the Kinect, a commercially available infrared position detection system. J Shoulder Elbow Surg.

[76] Poewe W, Seppi K, Tanner CM, Halliday GM, Brundin P, Volkmann J (2017). Parkinson disease. Nat Rev Dis Primers.

[77] Rosenbaum P, Paneth N, Levinton A, Goldstein M, Bax M, Damiano D (2006). The definition and classification of cerebral palsy. NeoReviews.

[78] Walther S, van Harten PN, Waddington JL, Cuesta MJ, Peralta V, Dupin L (2020). Movement disorder and sensorimotor abnormalities in schizophrenia and other psychoses-european consensus on assessment and perspectives. Eur Neuropsychopharmacol.

[79] Walther S, Ramseyer F, Horn H, Strik W, Tschacher W (2014). Less structured movement patterns predict severity of positive syndrome, excitement, and disorganization. Schizophrenia Bull.

[80] de Belen RAJ, Bednarz T, Sowmya A, Del Favero D (2020). Computer vision in autism spectrum disorder research: a systematic review of published studies from 2009 to 2019. Transl Psychiatry.

[81] Horwitz E, Schoevers R, Ketelaars C, Kan C, Van Lammeren A, Meesters Y (2016). Clinical assessment of ASD in adults using self-and other-report: psychometric properties and validity of the adult Social Behavior Questionnaire (ASBQ). Research in Autism Spectrum Disorders.

[82] Galna B, Barry G, Jackson D, Mhiripiri D, Olivier P, Rochester L (2014). Accuracy of the Microsoft Kinect sensor for measuring movement in people with Parkinson’s disease. Gait & posture.

[83] Weinberger M. The rise and fall of Kinect: why Microsoft gave up on its most promising product. Bussinessinsider. 2018.

[84] Nishani E, Çiço B, editors. Computer vision approaches based on deep learning and neural networks: Deep neural networks for video analysis of human pose estimation. 2017 6th Mediterranean Conference on Embedded Computing (MECO); 2017: IEEE.

[85] Qiang B, Zhang S, Zhan Y, Xie W, Zhao T (2019). Improved convolutional pose machines for human pose estimation using image sensor data. Sensors.

[86] Andrade-Ambriz YA, Ledesma S, Ibarra-Manzano M-A, Oros-Flores MI, Almanza-Ojeda D-L (2022). Human activity recognition using temporal convolutional neural network architecture. Expert Systems with Applications.

[87] Wrench A, Balch-Tomes J (2022). Beyond the edge: markerless pose estimation of speech articulators from ultrasound and camera images using DeepLabCut. Sensors.

[88] Doosti B, Naha S, Mirbagheri M, Crandall DJ, editors. Hope-net: A graph-based model for hand-object pose estimation. Proceedings of the IEEE/CVF conference on computer vision and pattern recognition; 2020.

[89] Luo Y, Ou Z, Wan T, Guo J-M, FastNet (2022). Fast high-resolution network for human pose estimation. Image and Vision Computing.

[90] Tronick E, Als H, Brazelton T (1979). Early development of neonatal and infant behavior. Human growth.

